# Health Status and Social Networks as Predictors of Resilience in Older Adults Residing in Rural and Remote Environments

**DOI:** 10.1155/2016/4305894

**Published:** 2016-07-10

**Authors:** Christine McKibbin, Aaron Lee, Bernard A. Steinman, Catherine Carrico, Katelynn Bourassa, Andrea Slosser

**Affiliations:** University of Wyoming, 1000 E. University Avenue, Laramie, WY 82071, USA

## Abstract

*Purpose*. Health status and social networks are associated with resilience among older adults. Each of these factors may be important to the ability of adults to remain in rural and remote communities as they age. We examined the association of health status and social networks and resilience among older adults dwelling in a rural and remote county in the Western United States.* Methods*. We selected a random sample of 198 registered voters aged 65 years or older from a frontier Wyoming county. Hierarchical linear regression was used to examine the association of health status as well as social networks and resilience. We also examined health status as a moderator of the relationship between social networks and resilience.* Results*. Family networks (*p* = 0.024) and mental health status (*p* < 0.001) significantly predicted resilience. Mental health status moderated the relationship of family (*p* = 0.004) and friend (*p* = 0.021) networks with resilience. Smaller family and friend networks were associated with greater resilience when mental health status was low, but not when it was high.* Conclusion*. Efforts to increase mental health status may improve resilience among older adults in rural environments, particularly for those with smaller family and friends networks.

## 1. Introduction

Older rural adults comprise a large and increasing percentage of the population in the United States. As of 2014, more than 16% of the population aged 65 years and older were categorized as living in rural settings by the US Census Bureau [1]. The proportion of older adults in rural areas has grown rapidly due in part to aging in place, retirement transitions, and outmigration of younger families [2]. Frontier areas are the most remote and sparsely populated areas among these rural settings. While it is well known that most older adults would prefer to age in their homes and communities [3], older residents in the most remote areas may be far from healthcare and social services, social venues, and other necessities to promote engagement and independent living. Additionally, transportation is frequently lacking and traveling to larger towns and cities where necessities including healthcare are located can be difficult [4].

Shifting demographic trends in rural and remote communities will bring societal changes and challenges. Primary among them will be identifying effective resources to foster health, wellbeing, and independence of older people as they face many of the stressors that often accompany aging in rural environments. For example, with increasing age, risk escalates for adverse outcomes associated with physical and mental health [5], disability [6], declining social network size [7], and economic security [8]. These stressors may be compounded by living in low-density population regions and can impact the quality of life of older adults, especially when the resources they may have available to help them adapt to change may be suboptimal.

The concept of resilience has gained traction as a means to explain how some individuals are able to bounce back from adverse events and stressors and adapt to their changing life circumstances, while others continue to struggle or decline in the face of similar events. On an individual level, resilience is thought to be a personality attribute that helps to neutralize negative effects of high stress [9]. According to Wagnild [10] resilience comprises dimensions of equanimity, a sense of purpose, perseverance, and acceptance of one's life, and a belief in one's self and capabilities. As a general resource possessed by many older adults, resilience can serve to buffer the negative impacts of stress, promote adaptation to late life changes, and contribute to maintenance of independent functioning and wellbeing [11].

As a research construct, resilience has received increasing attention in recent years. For instance, resilience has been examined in relation to specific age-related stressors, including pain [12, 13], mental health outcomes [14], aging with HIV/AIDS [15], bereavement following loss of a spouse [16], and having low income [10]. These studies suggest that resilience may play an important role in promoting healthy response to age-related change.

Much of the previous research on resilience has focused on urban and suburban samples [17] and few studies have examined resilience of community-dwelling older adults in rural and remote settings. One exception is research by Wells [18], which examined resilience levels of older adults residing in rural New York as well as predictors of resilience levels. Wells found that resilience levels were high in participants of this study. Importantly, resilience was not associated with sociodemographic factors and was only weakly associated with social networks. Significant relationships were also reported between physical and mental health status and resilience. While this was one of the first studies examining resilience among rural adults, the study was conducted in one rural community in New York. It is not clear to what degree these results will generalize to other rural communities or to older adults living in not only rural but also remote areas. Moreover, several studies show relationships between social networks and health among older adults [19–21]. Additionally, the work of Li and Zhang showed that relationships between social networks and health among older adults may be bidirectional such that those who have poorer health rely on more restricted social networks [20]. To date, few studies have been conducted to examine whether health status may moderate the relationship between social networks and other constructs like resilience among older adults, especially those residing in rural or remote areas.

The purpose of this study was to extend this small but important body of research. As part of this study, we examined the relationships between resilience and both social networks and health status, in a sample of older adults living in a rural environment. This study differs from the previous work, in that we sampled a population that is relatively more remote than the sample analyzed by Wells [18, 22] and substantially more remote than many other areas in the United States. The Western US state of Wyoming is unique with regard to its topography and the distribution of its population. Cities and towns are widely dispersed across the state's 97,093 square miles and long distances between points of interest are common. Additionally, the population of the state is very small relative to other states. According to estimates produced by data from the American Community Survey (ACS), there were just fewer than 600,000 Wyoming residents in 2014, equating to only about 5.8 persons per square mile (compared to 87.4 persons per square mile, nationally) [1]. Thus, many older Wyoming residents live in an expansive frontier environment that creates unique challenges for them with respect to accessing resources and necessities. Additionally, residents in general may live long distances away from their families, friends, and neighbors, potentially reducing the support and resources available when stressors such as acute and chronic illnesses arise. Given these circumstances, it is reasonable to expect that health outcomes and individual characteristics such as resilience may differ in this type of environment, even compared to other areas that are also categorized as rural. We expected that, like the work of Wells [18], social networks and health status would predict resilience. To build upon existing research, we examined moderation effects of physical and mental health status on the relationship of social networks and resilience in rural, community-dwelling older adults. We hypothesized that both physical and mental health status would moderate the relationship between social networks and resilience.

## 2. Methods

### 2.1. Study Sample

The University of Wyoming's Institutional Review Board approved all study methods. We used random sampling methods to obtain a sample of older adults residing in a frontier Wyoming county. First, we used the United States Department of Agriculture (USDA) Economic Research Service's 2013 Rural-Urban Continuum Codes [23] to determine the rurality of all counties in the state. This scale ranges from 0 to 9, with nine being the most rural based on the degree of urbanization and proximity to metro areas. We chose Fremont County, which scores a 7 on the scale, as the basis for our sample (compared to a rating of 6 for the county analyzed in the study by Wells [18]). Fremont County is relatively more rural than the rest of Wyoming, with only 4.4 persons per square mile. A prospective pool of study participants over the age of 65 residing in Fremont County (*N* = 3,368) was identified using the 2009 voting registration electoral roll. From this population, a sample of 600 individuals (18%) was randomly selected to receive a mailed survey packet.

Mailings included four components: (1) a cover letter that described the project, (2) a consent form that explained the recipient's rights as a research participant, (3) a raffle entry slip that gave participants an opportunity to win a $50 gift card as incentive to participate, and (4) the survey instrument (described below). Postcards reminding prospective participations of our request were mailed two weeks after the initial mailing. Of the 600 packets that were sent, 80 (13%) were returned as undeliverable. Of the 520 successfully delivered surveys, 225 were returned with useable data, resulting in a return rate of about 43%. A total of 27 incomplete surveys with only partial demographic data completed were removed prior to analysis, yielding a sample of 198 respondents.

### 2.2. Measures

The survey comprised questionnaires assessing the following four components: (1) sociodemographics, (2) degree of resilience, (3) self-reported physical and mental health status, and (4) size and quality of social networks.

#### 2.2.1. Sociodemographic Questionnaire

The sociodemographic questionnaire included items that assessed the age, gender, race/ethnicity, educational attainment, marital status, and employment status of participants.

#### 2.2.2. Connor-Davidson Resilience Scale

We measured each participant's degree of resilience using the Connor-Davidson Resilience Scale (CD-RISC; [24]). The CD-RISC is a 25-item questionnaire that asks participants to rate their level of agreement over the last month with statements such as “*I am able to adapt when changes occur*” and “*I tend to bounce back after illness, injury, or other hardships*.” Each statement was rated on a 0 (not true at all) to 5 (*true nearly all the time*) scale. Item responses were summed to generate a total score, with higher scores indicating greater resilience. The CD-RISC has demonstrated convergent validity and good internal reliability (*α* = 0.89) in previous work [24] as well as in the current study (*α* = 0.92).

#### 2.2.3. Short-Form Revised Health Survey

We assessed the physical and mental health status of participants using all twelve items of the Short-Form revised Health Survey (SF-12v2). Items in this instrument ask participants to rate their general health and to describe how physical and mental health issues limit or interfere with their daily activities and social activities. Weighted-items scores were used to calculate a Physical Component Summary score and a Mental Component Summary score. The SF-12v2 provides norms-based scores for the Physical Component Summary and the Mental Component Summary scores that are standardized (population mean = 50; standard deviation = 10) with higher scores reflecting greater functioning in each domain [25].

#### 2.2.4. Lubben Social Network Scale-6

We used the abbreviated Lubben Social Network Scale-6 to assess the social networks and supports of participants [26]. The Lubben Social Network Scale-6 is comprised of two 3-item subscales, which assess the size and quality of family and friend social networks. Several items are designed to assess the number of regular social contacts. For example, respondents are asked, “*How many relatives do you see or hear from at least once a month?”* Responses to these items are made on a five-point scale ranging from 0 (*none*) to 5 (*nine or more*). Other items ask about frequency of contact with family and friends (e.g.,* “How often do you hear from the relative with whom you have the most contact?”*). Responses to these items range from 0 (*less than monthly*) to 5 (*daily*). Finally, the quality and availability of social relationships are addressed using items such as “*How often is one of your relatives available for you to talk to when you have an important decision to make?*” Responses to these items range from 0 (*never*) to 5 (*always*). Higher scores on the Lubben Social Network Scale reflect more substantial social networks. Subscale scores less than or equal to 6 are suggestive of marginal family and friendship ties, and total scores less than or equal to 12 are indicative of social isolation. Both subscales have demonstrated convergent validity with measures of emotional support and social engagement [26]. The family and friend subscales have demonstrated good internal reliability in previous work (i.e., *α* = 0.89 and 0.82, resp.) [26] as well as in the current study (*α* = 0.80 and 0.81, resp.).

### 2.3. Statistical Analysis

All analyses were conducted using Statistical Package for the Social Sciences (SPSS) Version 22.0 [27]. Descriptive statistics (i.e., percentages, means, and standard deviations) were calculated to characterize the sample. Bivariate analyses were used to assess the zero-order correlations among SF-12vs component scores (i.e., Physical Component Summary and Mental Component Summary score), Lubben Social Network Scale subscale scores (family and friends), and resilience scores. A two-step hierarchical linear regression was used to simultaneously examine Physical Component Summary scores, Mental Component Summary scores, family network scores, and friend network scores as predictors of resilience while controlling for relevant sociodemographic variables. We entered age, gender, educational attainment (i.e., ≤ high school = 0; > high school = 1), marital status (i.e., single, divorced, or widowed = 0; married or long-term relationship = 1), and employment status (i.e., not employed = 0; employed = 1) in the first step of the model. In the second step of the model, we entered SF-12v2 Physical Component Summary and Mental Component Summary scores, as well as the family network and friend network scores. Tolerances were assessed for possible multicollinearity.

We used the PROCESS macro for SPSS to examine whether SF-12v2 Physical Component Summary scores and Mental Component Summary scores moderated the relationships between the social network subscales and resilience scores [28]. All predictors and covariates were mean centered. Heteroskedasticity consistent standard error estimators were used to guard against violations of homogeneity of variance [29]. Simple slopes were evaluated at one standard deviation above and below the mean of the moderator variable. The Johnson-Neyman approach was used to determine the boundary value of the moderator above and below which simple slopes were significantly different from zero [28, 30].

Bias-corrected and accelerated bootstrapping with 5,000 replications was used to estimate 95% confidence intervals (CI_95%_) for all correlation and unstandardized regression coefficients. Alpha was set to *p* < 0.05, and all tests were two-tailed.

## 3. Results

Table 1 depicts the result of descriptive analyses. The majority of survey respondents were female, non-Hispanic White, married, and retired. Age of participants ranged from 64 to 95 years and the average age was about 74 years. The mean resilience score was near 80 and comparable to the average resilience (i.e., CD-RISC) scores for the US general population (M = 80.4) [24]. Average social network scores for family and friends were each over 8 and greater than the suggested cut-point of 6 used for identifying marginal family and friendship networks among older adults [26]. Using a cut-score of 6 or lower, approximately 17% of the sample had marginal family networks and 24% of the sample had marginal friend networks. Physical Component Summary scores and Mental Component Summary scores of study participants were each slightly higher on average than the established population means for adults aged 65 years and older (Physical Component Summary score, M = 43.73, and Mental Component Summary score, M = 53.15) [25].  Table 2 shows correlations among all key predictor and outcome variables. Notably, significant positive correlations were found between resilience and Physical Component Summary scores (*p* = 0.025), Mental Component Summary scores (*p* < 0.001), family networks (*p* < 0.001), and friend networks (*p* < 0.001).

Table 3 shows unstandardized regression coefficients, standard errors, and Δ*R* ^2^ values for the two-step hierarchical linear regression analysis. In step one of the analysis, the covariates (i.e., age, gender, educational attainment, marital status, and employment status) did not explain a significant amount of variance in resilience scores, and none of the variables independently predicted resilience scores. In the second step, the Physical Component Summary score, Mental Component Summary score, family network, and friend network scores accounted for a significant increase in explained variance in resilience scores. Whereas, the Physical Component Summary scores and friend networks were not significant predictors of resilience, both the Mental Component Summary score and family network scores significantly predicted resilience scores while controlling for sociodemographic variables.

**Figure 1:**
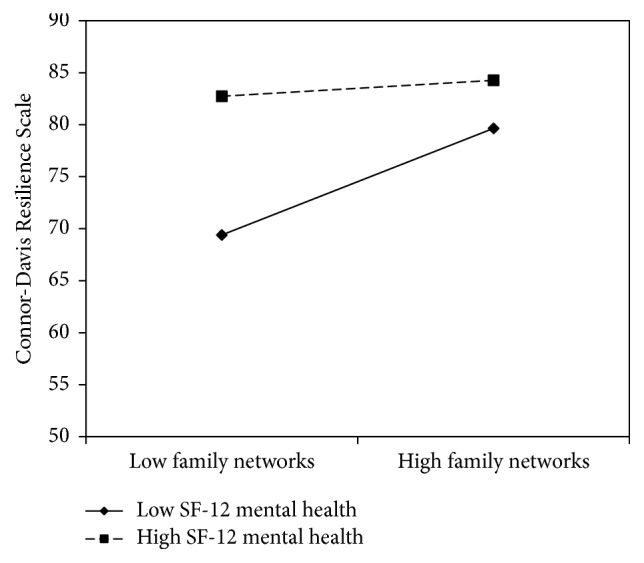
The simple slopes of Lubben Social Network Scale-6 family networks on Connor-Davis Resilience Scale scores at high and low levels of SF-12 Mental Health Component Summary scores.

As shown in Figure 1, the bootstrapped test of moderation yielded a significant interaction of family network by Mental Component Summary scores on resilience scores (*B * = −0.10, SE = 0.03, CI_95%_  [−0.17, −0.03], and *p* = 0.004), when controlling for age, gender, educational attainment, marital status, and employment status. Specifically, family network scores significantly predicted resilience scores at low (i.e., −1 SD) values of Mental Component Summary scores (*B* = 1.80, SE = 0.39, CI_95%_ [1.03, 2.57], and *p* < 0.001). However, no significant relationship between family networks and resilience was observed at high (i.e., +1 SD) values of Mental Component Summary scores (*B* = 0.27, SE = 0.42, CI_95%_  [−0.56, 1.10], and *p* = 0.529). Family networks significantly predicted resilience below, but not above, the Mental Component Summary score of 56.98. The majority of the sample (*n* = 117, 59.1%) scored below this boundary value.

**Figure 2:**
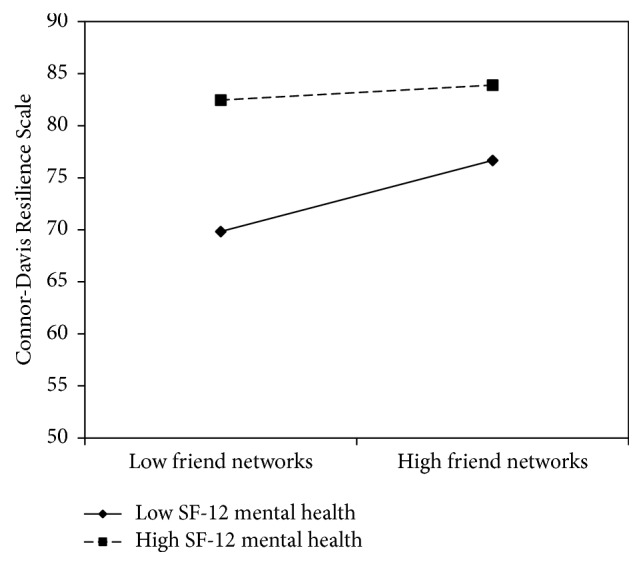
The simple slopes of Lubben Social Network Scale-6 friend networks on Connor-Davis Resilience Scale scores at high and low levels of SF-12 Mental Health Component Summary scores.

Figure 2 shows the significant interaction of friend networks by Mental Component Summary scores on resilience scores (*B* = −0.06, SE = 0.02, CI_95%_  [−0.10, −0.01], and *p* = 0.021), when controlling for sociodemographic covariates. Analysis of simple slopes showed a significant positive association between friend networks and resilience at low (−1 SD) levels of Mental Component Summary scores (*B* = 1.07, SE = 0.32, CI_95%_ [0.45, 1.71], and *p* = 0.001). There was no significant association between friend networks and resilience at high (+1 SD) levels of Mental Component Summary scores (*B* = 0.23, SE = 0.28, CI_95%_ [−0.33, 0.78], and *p* = 0.425). Friend networks were significantly associated with resilience below, but not above, the Mental Component Summary score of 49.45, below which most participants scored (*n* = 115, 58.1%).

Contrary to our expectations, moderation analyses did not show any additional significant interactions between the Physical Component Summary scores and family (*B* = −0.02, SE = 0.04, CI_95%_ [−0.09, 0.05], and *p* = 0.619) or friend (*B* = −0.05, SE = 0.04, CI_95%_ [−0.13, 0.02], and *p* = 0.163) networks when controlling for age, gender, educational attainment, marital status, and employment status.

## 4. Discussion

Developing a better understanding of resilience in rural older adults and its interrelationships with social networks and health status can help determine who may struggle to adapt successfully to stressful age-related changes and, ultimately, who will experience poorer outcomes. Literature shows that older adults living in rural and remote areas possess varying degrees of resilience, as do individuals living in urban and suburban areas [20]. For some older adults, a rural environment with strong social networks can provide valuable caring environments for people as they age. For others, rural environments may be isolating.

Results of our study showed that resilience levels of older adults living in a rural and remote county in Wyoming are generally high. Our results also suggest that both family social networks and mental health status are important predictors of resilience among these individuals. According to Fiori et al. [31], many types of social networks may contribute to relationship satisfaction in older adults and ultimately to better or worse health outcomes. For example, unmarried older adults often report lower physical and psychological wellbeing than married couples, and widowed women with friend-focused social networks report higher wellbeing than unmarried individuals. Older adults vary in the types and quantities of support needed from their social networks at different times. Utilizing networks in ways that serve the specific needs of older individuals is a likely contributor to the goal of successful aging via physical and psychological benefits, which also have potential to foster resilience [31]. With respect to social networks, results from this study suggest that family members are particularly important social resources that are associated with resilience among rural and remote older residents. In regions of the country where populations are widely dispersed, formal support resources are less likely to be directly on hand to provide assistance when it is needed. Thus, compared to more densely populated areas, family members of older adults living in remote areas may be required to step in more frequently when crises occur. It is possible that resilience can be fostered among residents in rural and remote communities by policies and programs that provide support for family members who may provide care for older residents.

Our results suggest that rural and remote, older persons with better functioning in the mental health domain also have higher degrees of resilience. This finding corresponds with the research of Fiori et al. [31], who reported similar outcomes. There are a few possible explanations for the association. First, resilience itself functions as an adaptive resource for coping with stressful situations. It may be that flexibility and adaptability serve as a buffer to stressors. Because our data were gathered at a single point in time, it did not take into account the order in which mental health-related issues arose relative to resilience status. Therefore, it is possible that our results reflect mental wellness that resulted from having high resilience, rather than the other way around. Second, older adults whose perceived mental health scores reflect higher levels of functioning may have traits that are more likely to promote and support the development of resilience. For example, older individuals who are able to maintain a positive outlook in the face of aging-related stressors can direct more of their cognitive resources toward productive problem-solving strategies and fewer resources toward monitoring and maintaining their mental health. Regardless of the explanation, our results suggest that encouraging mental health services and supporting access to them by older people in rural and remote areas can have a positive impact on resilience status and coping mechanisms.

Results of this study also expand the previous work of Wells [18] by suggesting that family social network may be an important moderator of the relationship between mental health functioning and resilience. A moderating relationship is one in which a variable—in this case family social networks—increases or decreases the magnitude of the relationship between two different variables [32]. Our findings suggest that strong family social networks can buffer the impact of poor mental health functioning with respect to its influence on resilience. In other words, when older rural and remote individuals experience mental health problems, strong family networks and the support they provide can help to make up the difference and restore resilience to levels near to those without mental health issues.

Dumitrache and colleagues [33] suggested that types of stressors and cultural contexts could impact the effects of social support in promoting resilience. Among older adults, factors associated with rural or remote living might strengthen, modify, or diminish relationships between social support, physical health status, and resilience. For example, Moore and colleagues [34] examined the interrelationships of perceived stress, social support, and self-reported successful aging and found that the influence of perceived stress on the relationship between physical functioning and self-reported successful aging was stronger among those with high levels of social support. Among rural and remote older adults in our study, physical health status did not predict resilience as it did in the study by Wells [18] and, thus, did not moderate the relationship between social support and resilience. It could be that the influence of the rural, Western context of participants in this study had a different influence compared to the rural Eastern context in which Wells' participants were recruited. One variable that may differ between the two samples is perceived stress. While neither this study nor the study by Wells [18] included a measure of perceived stress, this construct may have influenced relationships among the variables in both studies. Additional work that incorporates other potentially influential variables such as perceived stress and coping strategies might lead to greater understanding of the relationships between these variables.

This study is important to consider within the context of several limitations. First, like Wells [18, 22], we used mailed surveys to collect our data. Employing self-reported responses acquired through a mailed survey may not have captured the actual average level of resilience in rural and remote community-dwelling older adults. Selection bias may have limited participation by individuals with lower levels of resilience. The response rate in this study was 43%. While this rate was good, those with better health status, social networks, or resilience may have been more likely to respond. Additionally, although surveys were printed in 14-point font, participants with low visual acuity, chronic conditions, or low literacy may have been less able and less likely to respond to the survey. If this were the case, then our results might not generalize to those populations. This study did not gather information on all nonresponders. Therefore, it is not possible to discern how health status, social networks, or resilience may have differed among responding and nonresponding groups. Third, while this study addressed size and quality of social networks, other related constructs such as social connectedness, isolation, and loneliness were not measured. Other social network-related constructs should be addressed in future research. Fourth, as noted above, our study design was cross-sectional and, therefore, it is not possible to know with certainty the order of effects that we reported (i.e., whether mental health status precedes resilience, or the other way around). Future research that utilizes longitudinal data to assess how associations between social networks, health dimensions, and resilience levels covary over time would improve understanding of causal associations between these constructs. Lastly, our study only included older adults residing in rural and remote areas. Therefore, comparison of resilience and relationships of social networks and health status to resilience between rural and urban samples was not possible. Work by Wells [22] showed that resilience levels of older adults did not statistically differ depending on whether participants lived in rural, urban, or suburban areas. Thus, it is possible that there are no differences among older adults in these settings and that these results could generalize to older adults regardless of the size of their communities. However, it is also possible that older adults in rural versus urban settings utilize healthcare and mental health resources differently and/or that family and friend networks play unique roles in the lives of rural older adults compared to their more urban counterparts. Therefore, future work should include samples of rural, urban, and suburban residents to understand better the interplay of these constructs in each subgroup.

In addition to its statistical contribution, this study has clinical implications that are worthy of mention. The Institute of Medicine Report “Retooling for an Aging America: Building a Better Workforce” [35] echoes the recognized need for a workforce that is trained to meet the growing healthcare demands of an aging population. This report calls for an expansion of the roles of many members of the healthcare workforce including informal caregivers and direct care workers to improve care access [35]. While much attention has been given to mental health screening in primary care, other members of the workforce should also be prepared to identify mental health symptoms common among older adults. Moreover, all members of the healthcare workforce should have access to tools to assess social isolation among older adults and refer those individuals who are isolated or at risk for social isolation to community resources in order to prevent isolation or increasing isolation.

The inclusion of social supports in mental health interventions may also serve in mental health access and improve outcomes among older individuals facing mental health challenges. While collaborative care models have shown success for improving mental health outcomes, modifications of these models to include peer specialists to assist with transportation and delivery of basic interventions have shown success in improving access to care within small practices, such as those found in rural areas [36]. The delivery of socialization interventions by way of telehealth is another strategy to improve access to mental health services as well as health outcomes. For example, recent work by Choi and colleagues showed that tele-problem-solving therapy delivered through a Skype video call was an efficacious treatment for low-income homebound older adults [37]. Similarly, Jimison and colleagues [38] designed an intervention to increase social contact time of older adults in the home by enrolling them and a remote family member in a Skype-based health-coaching project. This program included weekly activities to meet target socialization goals as part of an individualized plan. Whereas positive impacts of telehealth interventions implemented to increase contact with social networks are feasible, additional challenges persist, such as lack of adequate technological infrastructure in rural areas to support cutting edge advancements that older adults in more urban regions may take for granted. Thus additional studies are needed to examine the specific needs and resources of older rural residents and their communities and the efficacy of technology for building strong social networks and improving mental health and resilience outcomes among rural and remote older adults.

## Figures and Tables

**Table 1:** Descriptive statistics for covariates, independent variables, and dependent variable (*N* = 198).

	M (SD)	*n* (%)
Sociodemographic		
Age	73.68 (6.90)	
Gender (female)		104 (52.5)
Race (white)		186 (93.9)
Non-Hispanic		198 (100)
Education (> high school)		149 (75.3)
Not employed		151 (76.3)
Married or having a long-term relationship		121 (61.1)

SF-12 health status		
MCS	53.49 (7.56)	
PCS	46.36 (10.50)	

Lubben Social Network Scale-6		
Friends	8.93 (2.85)	
Family	8.30 (3.18)	

CD-RISC	81.56 (12.23)	

*Note*. MCS: Mental Component Summary score; PCS: Physical Component Summary score; family: family network size; friend: friend network size; CD-RISC: resilience.

**Table 2:** Correlations among all independent variables and dependent variable (*N* = 198).

	PCS	Family	Friend	CD-RISC
MCS	0.11	0.21^†^	0.22^†^	0.48^‡^
PCS		0.08	0.14	0.16^*∗*^
Family			0.56^‡^	0.33^‡^
Friend				0.28^‡^

^*∗*^ *p* < 0.05; ^†^ *p* < 0.01; ^‡^ *p* < 0.001.

*Note*. MCS: Mental Component Summary score; PCS: Physical Component Summary score; family: family network size; friend: friend network size; CD-RISC: resilience.

**Table 3:** Regression results of resilience status on covariates and independent variables (*N* = 198).

	*B*	SE	Δ*R* ^2^	*p*
*Step 1*			<0.01	0.994
Age	−0.01	0.15		0.974
Gender	0.51	1.81		0.786
Relationship status	0.67	1.95		0.726
Employed	−0.31	2.00		0.876
Education	0.77	2.15		0.715

*Step 2*			0.29	<0.001
SF-12 MCS	0.67	0.11		<0.001
SF-12 PCS	0.10	0.08		0.165
LSNS-6 friend	0.23	0.29		0.424
LSNS-6 family	0.88	0.39		0.024

Full-model			0.30	<0.001

*Note*. MCS: Mental Component Summary score; PCS: Physical Component Summary score; LSNS-6 friend: friend network size; LSNS-6 family: family network size; CD-RISC: resilience.
